# Rapamycin treatment ameliorates HLA-B27-mediated gut inflammation and alters the microbiome in experimental spondyloarthritis

**DOI:** 10.3389/fimmu.2026.1755132

**Published:** 2026-03-20

**Authors:** Jinny Van Doorn, Stephen R. Brooks, Francesca LiCausi, Kelly Zhou, Naga S. Betrapally, Eva Gubitz-Hess, Antony Cougnoux, Stefania Dell’Orso, Shamima Islam, Robert A. Colbert, Fatemeh Navid

**Affiliations:** 1 Pediatric Translational Research Branch, National Institute of Arthritis and Musculoskeletal and Skin Diseases (NIAMS), NIH, Bethesda, MD, United States; 2 Biodata Mining and Discovery Section, NIAMS, NIH, Bethesda, MD, United States; 3 Laboratory of Integrative Cancer Immunology, Center for Cancer Research (CCR), NCI, National Institutes of Health (NIH), Rockville, MD, United States; 4 Section on Molecular Dysmorphology, NICHD, NIH, Bethesda, MD, United States; 5 Genomic Technology Section, NIAMS, NIH, Bethesda, MD, United States

**Keywords:** gut inflammation, HLA-B27, IL-17, rapamycin, spondyloarthritis

## Abstract

**Objective:**

To determine whether rapamycin affects HLA-B27-mediated gut inflammation in experimental spondyloarthritis (SpA).

**Methods:**

HLA-B27/human β_2_-microglobulin transgenic (B27-Tg) rats with gut inflammation were treated with rapamycin (1.5 mg/kg intraperitoneally, 3 times a week) or vehicle for 5 weeks. Healthy age-matched wild type (WT) rats were treated in parallel. Gut inflammation was evaluated via stool scoring and histological assessment. Transcriptome and microbiome analyses were performed on colon tissue and cecal luminal contents, respectively. Bulk immune cells were isolated from the colonic lamina propria of B27-Tg and WT animals, treated with rapamycin *ex vivo*, and pro-inflammatory cytokine expression was measured using qPCR.

**Results:**

Rapamycin treatment reduced stool and colon histological scores in B27-Tg rats compared to vehicle-treated B27-Tg controls. Transcriptome analysis revealed that rapamycin reduced expression of key pro-inflammatory cytokines like *Il17a, Il17f*, *Tnf*, *Il1a*, *IL1b*, and *Il22* in B27-Tg colon tissue compared to vehicle-treated B27-Tg controls. *Ex vivo* treatment of bulk immune cells isolated from B27-Tg rat colon with rapamycin reduced expression of *Il17a, Il17f*, *Ifng*, and *Il22* compared to vehicle-treated cells. Rapamycin treatment decreased the abundance of cecum microbiota associated with inflammation in B27-Tg rats. Rapamycin also altered the gut microbiome in WT rats, without associated changes in the tissue transcriptome.

**Conclusion:**

Our study demonstrates that rapamycin treatment substantially reduces HLA-B27-mediated gut inflammation in experimental SpA. Results from this pre-clinical model suggest further evaluation of rapamycin as a therapeutic strategy in HLA-B27 associated diseases is warranted.

## Introduction

Spondyloarthritis (SpA) encompasses a group of immune-mediated inflammatory diseases characterized by inflammation at multiple sites (1). In addition to affecting peripheral and axial joints, approximately 60% of patients with ankylosing spondylitis (AS), the prototypical form of SpA, have coexisting gastrointestinal tract inflammation. Gut inflammation can be subclinical, or in up to 10% of AS patients manifest as co-existing inflammatory bowel disease (IBD) (2). Notably, the severity of gut inflammation in SpA patients correlates with arthritis progression, suggesting a link between these sites (2) and underscoring gut inflammation as an important target in the treatment of SpA.

HLA-B27 is the strongest genetic risk factor for SpA, and when expressed in rats along with human β_2_-microglobulin (hβ_2_m), causes gut and joint inflammation resembling human SpA (3). The precise phenotype of B27-Tg rats, including the onset, location, and severity of inflammation, is dependent on transgene copy number (HLA-B27 and hβ_2_m), environment, and genetic background (3, 4). For example, gut inflammation occurs spontaneously in high copy number B27-Tg rats (3), while arthritis is largely dependent on the introduction of additional hβ_2_m transgenes and can be accelerated by immunization with heat-killed *Mycobacterium tuberculosis (*5). The SpA phenotype is also dependent on the presence of commensal gut microbiota (6, 7) and is associated with gut microbial dysbiosis that differs considerably between rat strains and environment despite common pathways of immune dysregulation (8). Thus, HLA-B27/hβ_2_m transgenic (B27-Tg) rats have served as an experimental model for investigating the mechanisms driving gut and joint inflammation and potential therapeutic approaches in SpA (4).

CD4+ T cells are implicated in mediating HLA-B27-associated disease in humans as well as rats (1, 3). B27-Tg rats exhibit expansion and activation of Th17 cells in the inflamed gut and joints (9, 10), which may be due in part to dendritic cell abnormalities (10), and recent evidence suggests that CD4+/CCR6+ Th17 cells expressing IL-17A and TNF are directly arthritogenic (11). Early evidence from this animal model contributed to the development of IL-17A inhibitors for treatment of AxSpA (12, 13).

Rapamycin, a potent inhibitor of the mechanistic (or mammalian) target of rapamycin (mTOR), is a well-established immunomodulatory drug approved for the prevention of post-transplant organ rejection (14). In addition to activating autophagy in cells, rapamycin suppresses Th17 cell differentiation and reduces IL-17A production (15). We have shown previously that rapamycin enhances the autophagic degradation of misfolded HLA-B27 in bone marrow–derived macrophages from B27-Tg rats (16). Rapamycin has been shown to be effective in preventing and attenuating both peripheral and axial arthritis in the immunization-induced model of SpA in low copy number B27-Tg rats (17).

Here, we investigated whether rapamycin has an impact on gut inflammation in high copy number B27-Tg rats that develop colitis and typhlitis. We show that rapamycin treatment dramatically reduces gut inflammation and nearly restores transcriptional profiles in B27-Tg rats to those of wild-type (WT) controls. Pro-inflammatory cytokine expression is almost normalized, and there are minimal effects in WT animals. Rapamycin reduces the relative abundance of pro-inflammatory microbes previously linked to gut inflammation in B27-Tg rats. Interestingly, it also alters cecal microbiota composition independently of HLA-B27 and effects on inflammation. Together, these findings show that rapamycin effectively attenuates HLA-B27-mediated gut inflammation in experimental SpA.

## Methods

### Animals

Hemizygous HLA-B*27:05 and human β_2_m-transgenic (HLA-B27-Tg) Lewis rats carrying 55 copies of HLA-B27 and 66 copies of human β_2_m in the 33–3 locus were used for this study. Aged-matched wild type (WT) Lewis rats were used in parallel. A total of 19 WT rats and 24 B27-Tg rats were used for the *in vivo* experiments, and an additional 4 WT animals and 9 B27-Tg animals for *ex vivo* studies. Rats were bred and housed in Association for Assessment and Accreditation of Laboratory Animal Care (AAALAC) approved facilities on the NIH Bethesda campus. Carbon dioxide (CO_2_) was used for animal euthanasia in accordance with the guidelines for euthanasia of rodents using CO_2_. Briefly, animals were placed in a 10-liter chamber and exposed to CO_2_ using a flow rate of 3–7 liters per minute, achieving a CO_2_ displacement rate of 30-70% per minute, until respiration ceases. Death was confirmed using accepted methods. All animal experiments were approved by the Animal Care and Use Committee at the National Institute of Arthritis and Musculoskeletal and Skin Disease (NIAMS).

### Stool scoring

Throughout the treatment course, animals were evaluated weekly for diarrhea via a standard stool scoring system. Stools from each animal were evaluated at treatment initiation and once weekly thereafter. Stools were assigned a score of 0 for solid stools, 1 for soft stools, 2 for semiliquid stools, and 3 for watery stools by veterinary facility staff.

### Histologic evaluation

For histologic evaluation, tissue was obtained from the distal colon or from the apex of the cecum of each animal. Tissue was obtained from vehicle-treated WT rats (n=9) or B27-Tg rats (n=11), as well as rapamycin-treated WT rats (n=10) and B27-Tg rats (n=13). Tissue was paraffin embedded, sectioned into 2–4 unique sections and stained with hematoxylin and eosin (H&E). Each tissue section was given a histological score in a blinded fashion by two independent observers according to a previously published scoring system (18). These scores were then averaged together to obtain the final histological score for the given animal. The tissue was evaluated for four separate categories: the presence or absence of gut-associated lymphoid tissue (GALT), degree of goblet cell loss, immune cell infiltrate, and area of tissue affected. Goblet cell loss, inflammatory infiltrate, and percent inflammation was scored from 0-3. The GALT category was given a score of 0 or 1 to indicate the presence or absence of GALT within a tissue slice. The scores for each subcategory were then summed together obtain a final composite histological score (histoscore).

### Colon tissue transcriptome analysis

Distal colon tissue samples obtained from rats (female and male) were homogenized in TRIzol reagent (Thermo Fisher Scientific). RNA was isolated using a standard phenol-chloroform protocol. RNA quantity and quality was assessed (Agilent) and RNA with an RNA integrity number (RIN) >7.0 was used for all RNA-seq analyses. Libraries were prepared according to the manufacturers guide (Illumina). The Illumina Novaseq 6000 system was used to perform 50-base, paired-end sequencing. Raw data were mapped to a customized genome based on rat rna6 with human transgenes HLA-B27 and hβ_2_m added to account for their transgenic expression in the experimental model. Partek Genomics Suite 7.0 was used to calculate Reads per Kilobase Million (RPKM) and to analyze differential gene expression via ANOVA. For further analysis, only genes where at least one sample had a max expression of RPKM > 1, coefficient of variance (C.V.) > 0.3, and a minimum of |2-fold| change, and p<0.05 were used. Partek Genomics 7.0 was also used for principal component analysis (PCA), and for generating hierarchical clustering heat maps and Volcano plots.

### Isolation of lymphocytes from colon tissue

Immune cells were isolated from colon tissue as described previously with some modifications (19). For these experiments 4 WT animals and 9 B27-Tg animals were used. Briefly, after removing connective tissue, colon tissue was washed in PBS several times and then cut into 0.5 cm sections in Hank’s balanced salt solution (HBSS, without Ca^2+^/Mg^2+^, Thermo Fisher Scientific) supplemented with 5% FBS and 1% Antibiotic-Antimycotic (Anti-Anti) (Thermo Fisher Scientific). To detach mucosa from the epithelial layer, sections were incubated in 1 mM DTT in HBSS supplemented with 5% FBS for 20 min at 37°C with shaking (120 RPM). Supernatants were removed and tissues were vortexed briefly and then placed in 5% FBS/1% Anti-Anti HBSS containing 300 U/mL collagenase-II (Worthington Biochemical) for 40 min at 37°C with shaking. Cells were pelleted, resuspended in supplemented HBSS, followed by gradient centrifugation with Lympholyte (Cedarlane) to collect the mononuclear fraction. Cells were then either plated in complete RPMI (10% FBS) for *ex vivo* experiments and treated as described or lysed in TRIzol reagent (Thermo Fisher Scientific) for RNA isolation. RNA was then isolated with the Zymo Direct-zol RNA miniprep kit, according to manufacturer’s protocol.

### Gene expression analysis

Total RNA was reverse transcribed with the iScript cDNA Synthesis kit (Bio Rad), according to manufacturer’s guidelines. To determine relative gene expression, quantitative PCR (qPCR) was performed via commercially available Taqman Assay (Thermo Fisher Scientific) primers for rat *Il23a* (Rn00590334_g1), rat *Il17a* (Rn01757168_m1), *Il17f* (Rn01757244_m1), *Il1a* (Rn00566700_m1), *Il1b* (Rn00580432_m1), Il22(Rn01766097_m1), with rat *Actb* (Rn00667869_m1) as the reference gene. The qPCR was performed using an QuantStudio 6 Flex.

### Immune signature analysis

Immune signature analysis as previously described (20) with some modifications. Homologous gene signature IDs were translated from rat to human using the UPPER function in excel. Duplicate gene IDs were then removed, with the duplicate with the greatest average expression (RPKM) kept. The ImSig program was then performed on the RNA-seq transcriptome for each animal. A conservative correlation threshold (r = 0.7) was used for feature selection and immune signature analysis (ImSig R package, version 1.0.0).

### Microbiome 16S rRNA sequencing

Luminal DNA was isolated from cecum and colon using DNeasy Kit (Qiagen, Valencia, CA) according to manufacturer’s instructions. 16S rRNA genes were amplified using primers as specified by the EMP sequenced on Illumina MiSeq. The greengenes database was used for alignment. Data were quality-filtered using quantitative insights into microbial ecology 2 (QIIME2) and taxa were summarized to kingdom, phylum, class, order, family, genus, and the species level (Phylogenetic Level 7*).* QIIME2 was used to visualize PCoA plots for beta diversity. Differential abundance was assessed for magnitude and significance using linear discriminant analysis (LDA) effect size (LefSe) analysis. We compared relative abundance of the gut microbes (|LDA|>2, p <0.05) in rapamycin-treated with their wild-type and vehicle-treated controls in the cecal and colon lumen.

### Interomics

Host transcriptome cecum and colon gene expression data was correlated with 16S rRNA sequencing of the respective colon and cecum lumen, as previously reported (21) with slight modifications. Pearson’s correlation coefficient (r) was calculated and significant values (p <0.001) were subjected to hierarchical clustering. Microbes correlating with at least 5% of the total transcripts and transcripts that correlated with least of 4 microbes (2.5% of total) are shown.

### Statistical analysis

One-way parametric ANOVA in GraphPad Prism Version 10.3.1. (LaJolla, CA) was used to assess differences between means, with *p* < 0.05 considered statistically significant (*). Data was assessed for normality (Kolmogorov-Smirnov’s test) prior to using a one-way parametric ANOVA (Tukey’s multiple comparisons). For non-normally distributed data, a Kruskal Wallis test was used (Dunn’s multiple comparisons). For transcriptome analysis, differentially gene expression was evaluated using ANOVA on log-2 transformed RPKM data in Partek Genomics Suite as described in the method section above. Unless otherwise stated, the figures show three independent experiments. Simple linear regressions were performed to assess linear correlation in GraphPad prism, with the 95% confidence interval shown, or in Partek Genomics Suite (Pearson’s correlation) for the inter-omics analysis as described in the methods section above.

## Results

### Rapamycin treatment reduces gut inflammation

**Figure 1:**
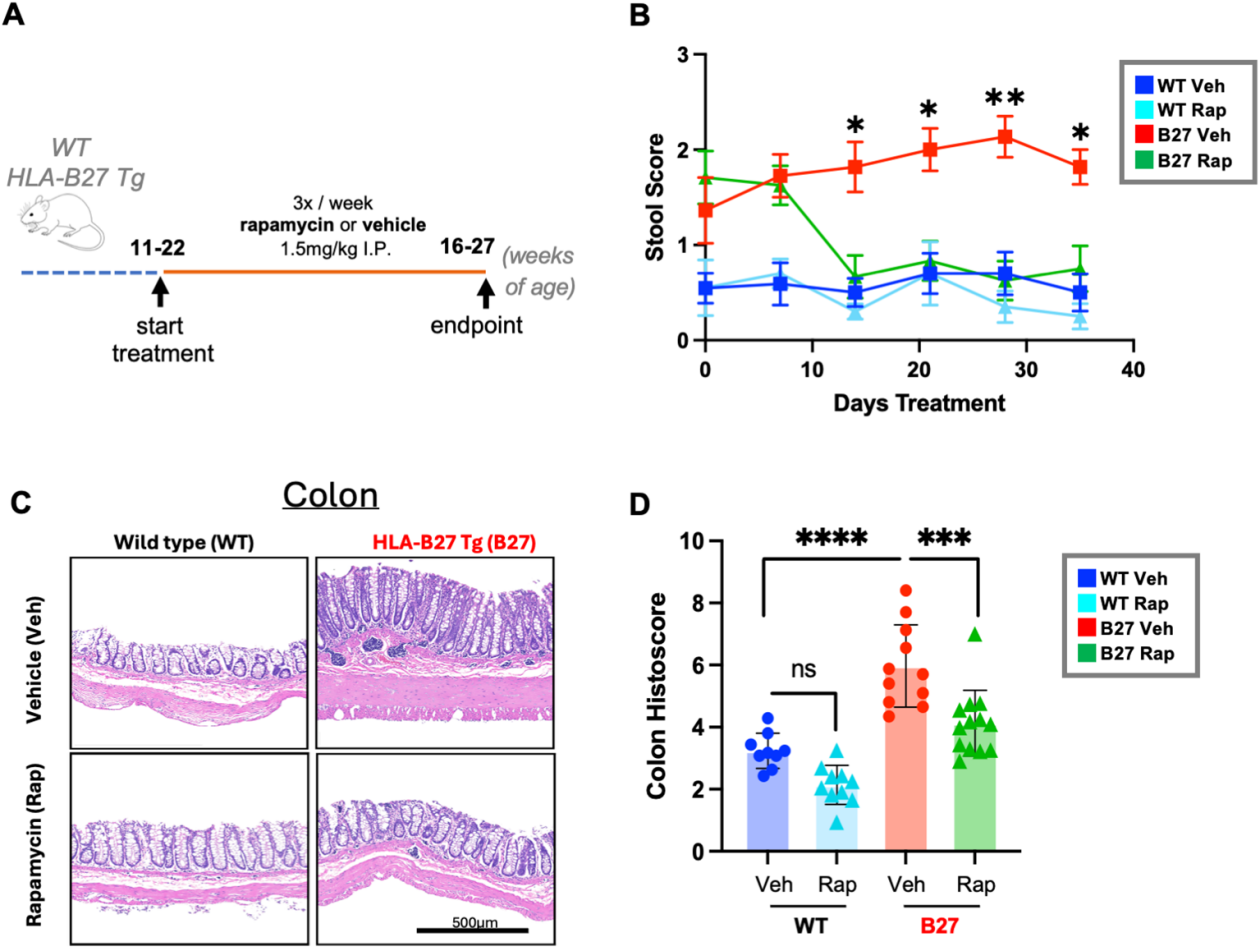
Rapamycin treatment reduces HLA-B27-mediated gut inflammation. **(A)** Experimental design. Rapamycin treatment was initiated between 11 and 22 weeks of age. Animals were injected intraperitoneally (i.p.) with 1.5 mg/kg rapamycin or vehicle 3 times a week for 5 weeks (n = 9–13 animals per treatment group, with approximately equal numbers of male and female rats). Age-matched WT (non-transgenic) animals were treated in parallel as controls. **(B)** Animals were evaluated for gut inflammation via stool scores. Each data point represents an average of stool scores in the respective group at the specified timepoint with mean +/- SEM. Statistical analysis was performed via non-parametric Kruskal Wallis multiple comparisons tests. (*p < 0.05, **p < 0.01). **(C)** Representative images (6x magnification) of H&E-stained distal colon tissue. Tissue was collected from animals at the experimental endpoint. (scale bar: 500 µm). **(D)** Histology scores of H&E-stained distal colon tissue. Data points represent the average score from 2–8 tissue sections per animal. Bar shows the mean +/- SEM for all data points of a single treatment group. Statistical comparisons were performed via one-way parametric ANOVA (*p < 0.05, **p < 0.01, ***p < 0.001, ****p < 0.0001).

Gut inflammation is a major component of experimental SpA in high copy number B27-Tg rats. In our colony, inflammation generally begins at 8-weeks of age and worsens over time (8). To assess the effect of rapamycin on gut inflammation, 11 to 22-week-old B27-Tg rats and age matched WT animals were treated for 5 weeks with rapamycin or vehicle as described (5) (**Figure 1A**). Before (Day 0) and during the rapamycin treatment stool samples were collected and scored. B27-Tg rats exhibited elevated stool scores before the start of treatment, consistent with the presence of gut inflammation (**Figure 1B**). Stool scores were significantly reduced compared to vehicle-treated rats after two weeks of rapamycin treatment, and remained normal throughout the treatment period (**Figure 1B**). Rapamycin did not alter the stool scores of WT rats.

After 5 weeks of treatment, tissue samples were collected, and gut inflammation was assessed by histological scoring of the colon (**Figures 1C, D**) and cecum (**Supplementary Figures 1A, B**). Increased histological scores in vehicle-treated B27-Tg rats reflect loss of goblet cells, the presence of inflammatory cell infiltrates, and the relative amount (percent) of inflamed tissue (**Supplementary Figures 1C, D**). Rapamycin treatment reduced inflammation in B27-Tg rats significantly, consistent with the decreased stool scores (**Figures 1C, D**; **Supplementary Figures 1C, D**).

Together these data confirm that rapamycin treatment reduces gut inflammation in HLA-B27 Tg animals.

### Rapamycin treatment dramatically alters the colon transcriptome and reduces proinflammatory cytokine gene expression

**Figure 2:**
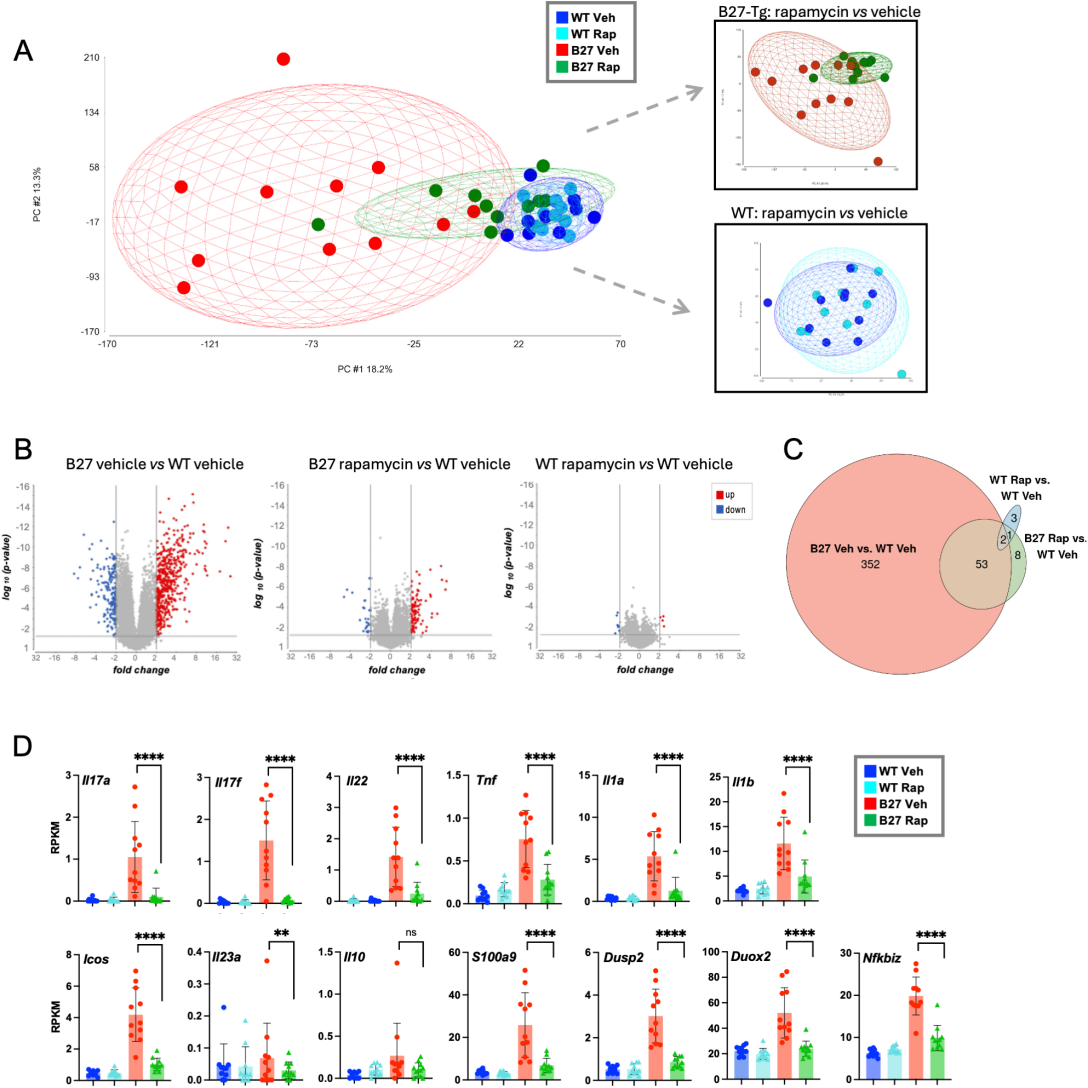
Rapamycin normalizes the colon transcriptome of B27-Tg rats. **(A)** Principal component analysis (PCA) using bulk RNA-seq of distal colon tissue. Vehicle-treated WT (dark blue); rapamycin-treated WT (light blue); vehicle-treated B27-Tg (red); rapamycin-treated B27-Tg (green). **(B)** Volcano plots of differential colon transcript expression between the indicated conditions (B27-Tg vehicle *vs.* WT vehicle; B27-Tg rapamycin *vs.* WT vehicle, and WT rapamycin *vs.* WT vehicle). An ANOVA statistical test was performed on log-2 transformed RPKM values, comparing the treatment groups as indicated. Genes that are significantly differentially expressed (p < 0.05 and |FC| > 2) between the two conditions are indicated on each plot [underexpressed (blue), overexpressed (red)]. **(C)** Euler diagram shows the overlap of differentially expressed genes between the groups shown in **(B)**. **(D)** Selected colon gene expression in individual animals. Statistical comparisons are as indicated. Statistical analyses were performed via ANOVA analysis on log-2-transformed RPKM values (**p < 0.01; ****p < 0.0001).

Next, we analyzed the effect of rapamycin on the gut transcriptome. Distal colon tissue collected at the experimental endpoint was analyzed for differences in gene expression via RNA-seq. Principal Component Analysis (PCA) of the transcriptome showed that samples from the B27-Tg vehicle-treated group (red) clustered distinctly from the vehicle-treated WT group (dark blue), with greater intra-group variability observed for B27-Tg animals (**Figure 2A**). Rapamycin treatment of B27-Tg rats (green) restored much of the transcriptome, driving the cluster to overlap substantially with the WT groups (dark and light blue), including a reduction in intra-group variation (**Figure 2A**; top inset). The colon transcriptome of WT animals was marginally affected by rapamycin treatment, indicating that in the absence of inflammation the effect of rapamycin on colon gene expression is minimal (**Figure 2A**; bottom inset).

Volcano plots were generated to visualize differentially expressed genes. Samples from vehicle-treated B27-Tg rat colon showed the highest level of differentially expressed genes when compared to the corresponding WT controls (**Figure 2B**, left). Rapamycin treatment of B27-Tg rats dramatically reduced the number of differentially expressed genes compared to vehicle-treated WT rats (**Figure 2B**, second panel) or compared to rapamycin-treated WT animals (**Supplementary Figure 2A**, left panel). For comparison, differentially expressed genes in rapamycin-treated B27-Tg rats compared to vehicle-treated B27-Tg rats are shown in **Supplementary Figure 2A** (right panel). It should be noted that rapamycin treatment of WT rats resulted in minimal gene expression differences compared to vehicle-treated controls (**Figure 2B**, third panel). The number of differentially expressed genes for each comparison and the overlap between groups is shown in the Euler diagram (**Figure 2C**), demonstrating that rapamycin is almost exclusively affecting gene expression in the inflamed B27-Tg colon tissue.

Further analysis of individual genes whose expression is reduced by rapamycin treatment shows near normalization of pro-inflammatory cytokines including *Il17a*, *Il17f*, *Il22*, *Tnf*, *Il1a, Il1b*, and *Il23a*, as well as other markers of gut inflammation, and *Nfkbiz*, an IkappaB family member that regulates the NF-κB pathway (**Figure 2D**). Analysis of the downregulated gene set (**Supplementary Figure 2A**, right panel, blue dots) revealed pro-inflammatory pathways, such as ‘inflammatory response’, ‘Th17 cell differentiation’, ‘T cell receptor signaling’, and ‘NF-kB and TNF-signaling’ (**Supplementary Figure 2B**). Immune signature analysis of the colon transcriptome revealed correlations between the relative abundance of T cells, B cells, NK cells, and macrophages, and histology scores in vehicle-treated B27-Tg rats and showed that rapamycin treatment reduced the relative abundance of each cell population except macrophages (**Supplementary Figure 2C**). These results correspond with the decrease in inflammatory infiltrate observed in rapamycin-treated B27-Tg animals compared to their corresponding vehicle-treated B27-Tg controls. Together, these data suggest that rapamycin treatment led to a major reduction in HLA-B27-mediated inflammation with reduced pro-inflammatory gene expression, without affecting the transcriptome of healthy WT animals.

### *Ex vivo* treatment of B27-Tg colon lymphocytes with rapamycin reduces proinflammatory cytokine expression

**Figure 3:**
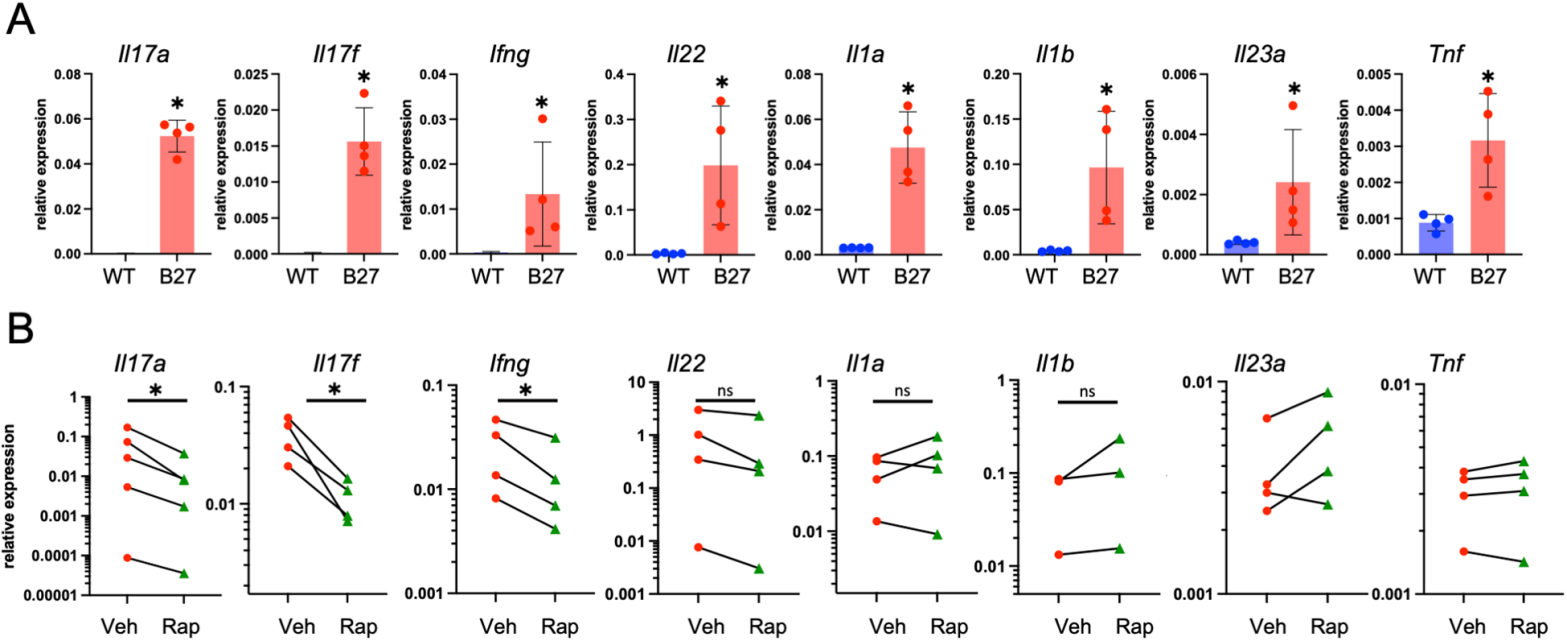
Cytokine expression in bulk immune cells and response to rapamycin *ex vivo*. **(A)** Colonic lamina propria immune cells were isolated from 5–6-month-old animals and assessed for cytokine expression. Each data point denotes the expression level in bulk immune cells from a single animal (n = 4). Significance was determined via Mann Whitney test (*p < 0.05). **(B)** Colonic lamina propria bulk immune cells isolated from 5–6-month-old B27-Tg rats were treated with rapamycin or vehicle for 22 h. Cells were collected, RNA was isolated and qPCR was performed for the indicated cytokines. *β*Act expression was used as a housekeeping gene for normalization. Each datapoint denotes cells isolated from a single animal (n = 3-5). Significance was determined via paired t-test (*p < 0.05).

To look for direct effects of rapamycin on cytokine expression, bulk immune cells were isolated from the colons of 5–6-month-old untreated B27-Tg and WT rats. Cytokine expression was measured using qPCR, and significantly increased expression of *Il17a*, *Il17f*, *Ifng, Il22*, *Il1a*, *Il1b*, *Il23a* and *Tnf* was found (**Figure 3A**), similar to whole tissue transcriptome results. Next, we treated isolated bulk immune cells from B27-Tg rats with 50 nM rapamycin or vehicle for 22 hours and measured expression of these cytokines. Rapamycin significantly reduced the expression of *Il17a*, *Il17f*, and *Ifng* (**Figure 3B**). Differences in expression of *Il23a, Il1a*, *Il1b*, and *Tnf* transcripts were not observed, and while *Il22* expression was lower in all treated samples, the difference was not statistically significant. These results confirm the strong effects of rapamycin on Th17 T cell cytokine expression (22) and support important roles for these cytokines *in vivo* in HLA-B27-mediated gut inflammation.

### Rapamycin treatment resolves dysbiosis

**Figure 4:**
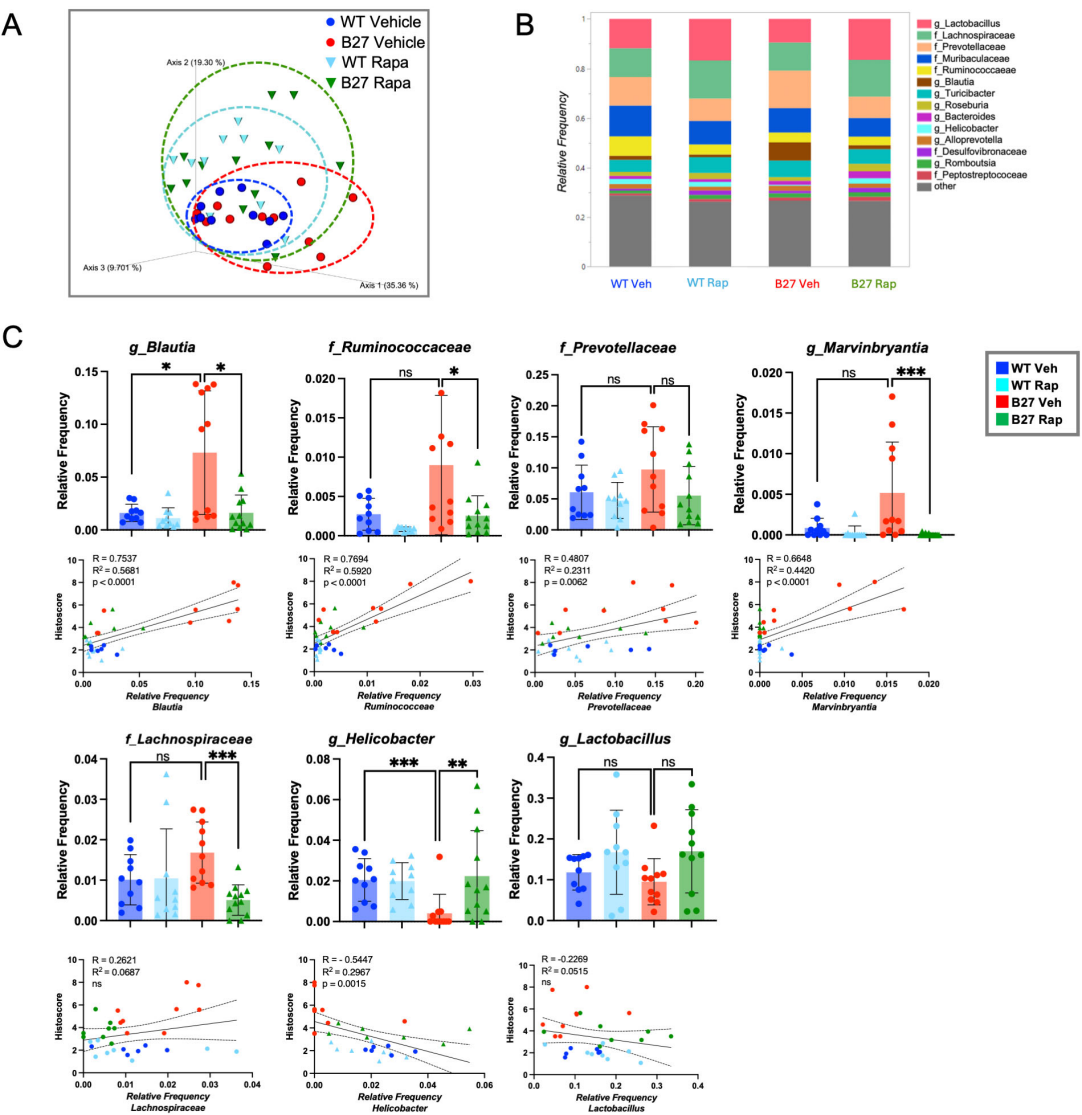
Microbial abundance is affected by rapamycin treatment. **(A)** Principal Coordinate Analysis (PCoA) of cecal microbial composition (beta diversity) of all four treatment groups. Each data point represents a cecum lumen sample collected from a single animal. The data distribution of each treatment group is shown by ellipsoids, with vehicle- and rapamycin-treated B27-Tg (red and green, respectively) and vehicle- and rapamycin-treated WT (dark blue and light blue, respectively) samples as indicated. **(B)** Stacked bar showing the relative frequency of most abundant microbes in cecum lumen. **(C)** Bars represent the mean frequency of selected microbes. Plots below each graph depict the relative frequency as a function of the cecum histology scores. Each data point represents results from a single animal. *p<0.05; **p < 0.01; ***p < 0.001.

We previously reported dysbiosis that correlated with inflammation in the colon and cecum of B27-Tg rats (8). To determine whether rapamycin affects HLA-B27-associated gut dysbiosis we analyzed the microbial composition of the cecum lumen using 16S rRNA sequencing. Principal Coordinate Analysis (PCoA) revealed that after rapamycin treatment, B27-Tg cecal lumen microbiota were more similar to rapamycin-treated WT rats (**Figure 4A**; **Supplementary Figure 3A**; dark green vs. light blue symbols) than vehicle-treated WT animals (**Figure 4A** dark blue symbols), suggesting independent effects of rapamycin on gut microbiota (i.e. effects not only secondary to the resolution of inflammation). This was confirmed by a direct comparison of the rapamycin-treated WT rats with WT vehicle-treated animals (**Figure 4A**; **Supplementary Figure 3A**; light blue vs. dark blue symbols). This indicates that not only does rapamycin treatment restore many of the changes associated with dysbiosis in B27-Tg rats, but it also alters the gut microbiome in the absence of inflammation (**Supplementary Figure 4**).

We next examined the effect of rapamycin on cecal microbes at the genus or family level (**Figures 4B, C**). The 14 microbes with the highest relative frequency in each group of rats are shown in **Figure 4B**. There is a notable increase in *Blautia* in vehicle-treated B27-Tg rats, which correlates positively with cecum histology scores (**Figure 4C**), confirming our previous findings (8). Other microbes showing elevated abundance and correlating with histology scores include *Ruminococcaceae*, *Marvinbryantia* and *Prevotellaceae* (**Figure 4C**). Rapamycin reduced the relative frequencies of *Blautia*, *Ruminococcaceae*, *Marvinbryantia*, and *Prevotellaceae*, although the latter was not statistically significant and was quite variable in non-inflamed cecum lumen (**Figure 4C**). Host transcripts significantly correlated with microbial abundance were subjected to hierarchical clustering, revealing several microbial taxa such as *Blautia*, *Prevotelleaceae*, *Marvinbryantia*, and *Ruminococceae*, correlating with pro-inflammatory genes such as *Il17*, *Il23*, and type II IFN signaling (**Supplementary Figure 3B**). This inter-omic analysis confirms and extends our previous findings (8).

Rapamycin treatment also significantly reduced the relative frequency of *Lachnospiraceae* which was modestly increased in inflamed B27-Tg gut (although not statistically significant) compared to the WT controls and correlated weakly with histological scores. Interestingly, *Helicobacter* was strongly reduced in B27-Tg cecal tissue and correlated negatively with cecal histological scores (**Figure 4C**). Rapamycin treatment restored *Helicobacter* in B27-Tg rats to the level seen in WT controls. *Lactobacillus* was slightly decreased in B27-Tg rats and correlated negatively with histological scores but was more abundant after rapamycin treatment of both WT and B27-Tg rats (**Figure 4C**). Together these data show rapamycin affects the abundance of microbes in B27-Tg cecum lumen which associate with inflammation, as well as having independent effects on microbial composition.

## Discussion

We demonstrate for the first time that rapamycin treatment substantially reduces gut inflammation in an experimental model of SpA. Stool scores in B27-Tg rats normalized within two weeks of initiating treatment and remained normal throughout the treatment period. After 5 weeks of rapamycin cecum and colon histology scores were significantly reduced, with a notable loss of inflammatory infiltrates and restoration of goblet cells. Close to 90% of tissue gene expression differences were eliminated by rapamycin treatment, and Th17 cytokines (*Il17a*, *Il17f*, and *Il22*), *Tnf*, *Il1a*, and *Il1b*, as well as *Il23a* were dramatically reduced or normalized. IL-1 and IL-23 are important drivers of Th17 expansion and cytokine production (23) and thus may be particularly important. Furthermore, rapamycin improved the balance of many microbes previously reported to be associated with gut inflammation.

To our knowledge this is the most effective treatment reported to date for gut inflammation in B27-Tg rats, aside from completely eliminating gut microbes (6, 7). TNF inhibition was reported to be effective at preventing the development of gut inflammation if administered by 9 weeks of age, while starting treatment at 18 weeks of age was ineffective (24). The TNF inhibition study was performed in Fischer rats (F344.33-3) carrying the same transgene locus used for our study (24). In our experience, F344.33–3 rats develop somewhat more severe intestinal inflammation than the Lewis animals (LEW.33-3) used here (8). The LEW.33–3 animals develop gut inflammation around 2 months of age (8), as reflected clinically by the elevated stool score (~1.5) (**Figure 1B**), which increases over the next month to about 2.0. Importantly, stool scores of 1.5-2.0 reflect soft to mushy stool that is still formed. Even with prolonged gut inflammation and higher stool scores, these rats do not lose weight and do not appear otherwise sick. Thus, this model may be comparable to many SpA patients with sub-clinical gut inflammation rather than overt IBD. While F344.33–3 rats could conceivably be more resistant to treatment, this seems unlikely given the overall similarities in pathogenesis reflected by similar colon and cecum gene expression patterns in F344.33–3 and LEW.33–3 rats (8). It should be noted that rapamycin has also been reported to be somewhat effective in treating chemical damage-induced colitis in mice (25–28), and in the *Il10*-deficient mouse model (29).

Rapamycin exerts its biological effects through inhibition of mTOR signaling (30). Since mTOR (primarily mTORC1) drives the expansion of T helper cells while inhibiting the development of regulatory T cells (Tregs), rapamycin is a potent inhibitor of Th17 development and IL-17 expression (31), and at the same time it promotes development of Tregs (30). In studies of mouse colitis, rapamycin has been shown to restore the Treg/Th17 balance (26), reduce leukocyte extravasation into the tissue (25), and improve intestinal barrier function (28). We demonstrated that rapamycin reduces the overexpression of *Il17a*, *Il17f*, and *Ifng* in bulk immune cells isolated from colon tissue of B27-Tg rats, consistent with reported effects on Th17 cytokine expression (31). *Il22* expression was also reduced, but the change did not reach statistical significance. The reduction in IL-17A expression is similar to what Chen et al. (17) reported for patient-derived PBMCs from SpA patients in their study showing the efficacy of rapamycin for peripheral and axial arthritis in the immunization model of HLA-B27-induced SpA. In contrast to effects on T cell cytokines, we did not see a reduction in the expression of *Il23a*, *Il1a*, *Il1b* or *TNF* in bulk colon immune cells treated with rapamycin *in vitro*. These cytokines may derive primarily from myeloid and epithelial cells, perhaps explaining resistance to direct effects of rapamycin. However, it should be noted that expression of *Il23a*, *Il1a*, *Il1b* and *TNF* (in addition to *Il17a*, *Il17f*, *Il22*) were reduced when measured in whole colon tissue from rapamycin-treated animals. This is consistent with the idea that the inhibition of T cell cytokines from Th17 and Th1 cells may interfere with a positive feedback loop that promotes tissue inflammation. Rapamycin might also be having a positive effect on epithelial barrier function (32) or leukocyte extravasation into the tissue (25) in B27-Tg rats, all of which could contribute to the resolution of gut inflammation in experimental SpA.

Previously, we identified gut microbes in the genera *Blautia* and *Prevotellaceae* as potential pathobionts associated with host immune dysregulation and activation of pro-inflammatory gene networks in experimental SpA (8, 21). In this study, we confirmed and extended these findings. Correlations between host cecal transcript expression and microbial taxa abundance showed that increased relative abundance of *Blautia*, *Prevotellaceae*, *Marvinbryantia*, and *Ruminococcaceae* were associated with transcripts involved in pro-inflammatory pathways including IL-17, IL-23, and Type II IFN signaling. The abundance of these taxa also loosely correlated with cecum histological scores, suggesting an association between host inflammation and microbial abundance. Whether the increased abundance of these microbes contributes to, or is a consequence of inflammation, cannot be determined from these data and further investigation would be necessary. The relationship is likely complex, involving signals between host and microbiota. In damage-induced colitis in mice, dextran sodium sulfate (DSS)-induced inflammation was associated with increased abundance of *Bacteroides sartorii* and decreased *Lactobacillus*, both of which were restored by rapamycin treatment (27). Interestingly, we observed a strong reduction of genus *Helicobacter* in inflamed B27-Tg cecum tissue, which negatively correlated with inflammation and was significantly increased after rapamycin treatment to levels similar to WT controls. Whether rapamycin exerts direct effects on microbial abundance or function that in turn are immunomodulatory remains at this point uncertain. Additionally, whether the independent changes of the microbiome by rapamycin observed in the healthy WT rats is playing a role also in inflammation is unclear (**Supplementary Figure 4**). The effect of rapamycin on the gut microbiome of healthy WT animals lacking inflammation is not surprising since the drug is an antibiotic that is excreted in the feces. Indeed, a recent study documented effects of rapamycin on certain bacterial genera in the gut lumen of healthy mice (33).

The results we report here demonstrate striking efficacy of rapamycin in suppressing gut inflammation in B27-Tg rats. Together with the previous demonstration that rapamycin limits the development and severity of axial and peripheral arthritis in the immunization-induced B27-Tg rat model (17), our results suggest that rapamycin is a strong candidate for a therapeutic trial in axial SpA. While IL-17A inhibitors are effective in axial SpA, their use is limited in patients with gastrointestinal inflammation as this cytokine has protective effects in the gastrointestinal tract regulating epithelial permeability and intestinal barrier function (34) and regulating certain microbial populations (35). Interestingly, rapamycin (sirolimus) was beneficial in a pediatric patient with TNF inhibitor-refractory ulcerative colitis (36). In this case report, sirolimus led to marked improvement in disease symptoms within six days, with clinical remission achieved within two months. It is intriguing that rapamycin, which almost certainly exerts some of its anti-inflammatory effects in the gut by reducing Th17 development and IL-17A production, is so effective in the B27-Tg rat models of experimental SpA which mimic key features of the human condition, and raises the possibility that rapamycin may be beneficial in axial SpA even in the context of active gut inflammation.

## Data Availability

The RNASeq data are deposited in the NCBI GEO repository, accession number GSE308302. Microbiome data are deposited in the NCBI BioProject repository, accession number PRJNA1333912.
