# Supplementary material for: Rapamycin treatment ameliorates HLA-B27-mediated gut inflammation and alters the microbiome in experimental spondyloarthritis
Source: Front Immunol. 2026 Mar 20;17:1755132. doi: 10.3389/fimmu.2026.1755132 (PMC13047741; doi:10.3389/fimmu.2026.1755132)
Supplement: Supplementary file 1 [file DataSheet1.pdf]

Supplementary Material

Rapamycin treatment ameliorates HLA-B27-mediated gut inflammation and alters the microbiome in experimental spondyloarthritis

Jinny Van Doorn[1], Stephen R. Brooks[2], Francesca LiCausi[1], Kelly Zhou[1], Naga S. Betrapally[3], Eva Gubitz-Hess[1], Antony Cougnoux[4], Stefania Dell'Orso[5], Shamima Islam[5], Robert A. Colbert[1*], Fatemeh Navid[1*‡]

***Correspondence:** Fatemeh Navid, PhD**,** fatemeh.navid@nih.gov

Supplementary Figures

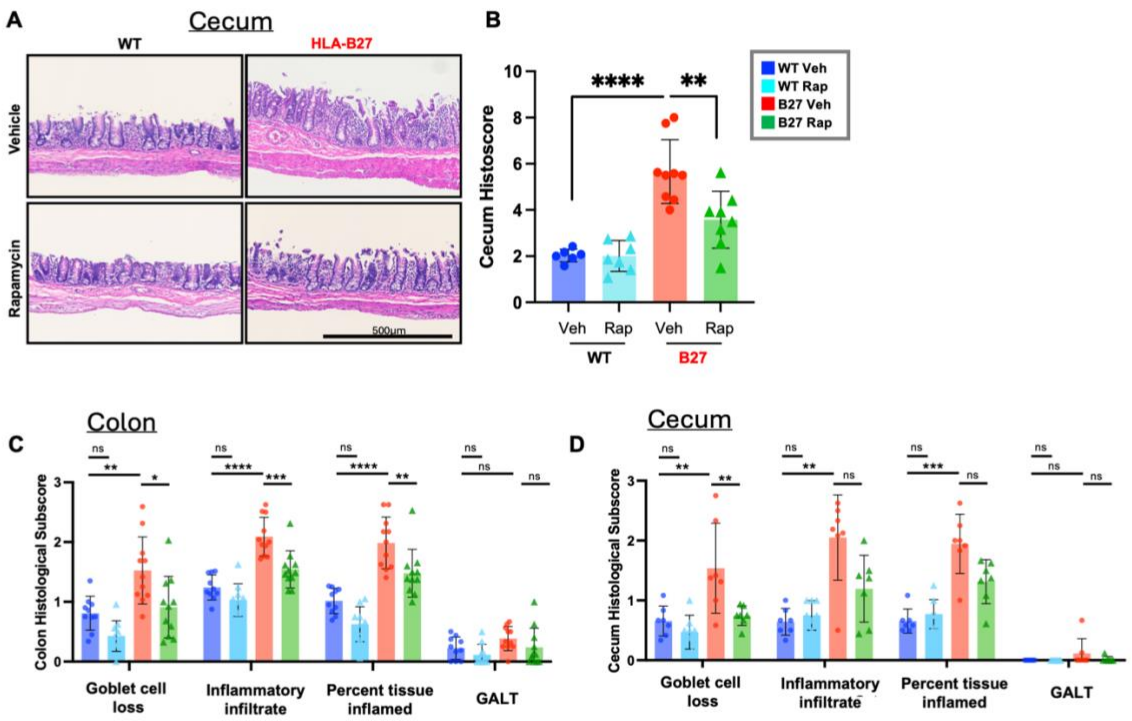

Supplemental Figure 1. Rapamycin treatment reduces cecum inflammation in B27-Tg animals. (**A**) Representative images (6x magnification) of H&E-stained cecum tissue sections used for histological scoring. This tissue was collected from animals at the experimental endpoint. scale bar: 500 μm. (**B**) Histology scores of H&E-stained cecum tissue collected from animals at experimental endpoint. Each data point represents the average score calculated from 2-8 slices from a single animal. Statistical comparisons were performed via one-way parametric ANOVA (**p < 0.01; ****p < 0.0001). (**C**) Colon histology scores of the single categories from data shown in Figure 1D: goblet cell loss, inflammatory infiltrate, precent tissue inflamed, gut-associated lymphoid tissue (GALT). Statistical comparisons were performed via one-way parametric ANOVA (with Tukey's multiple comparison's test). Normality was assessed via Kolmogorov-Smirnov test. The GALT sub-score did not pass the normality test, and statistical comparisons were performed with a nonparametric Kruskal Wallis test (Dunn's Multiple Comparisons) (*p<0.05; **p < 0.01; ***p < 0.001; ****p < 0.0001). (**D**) Cecum histology scores of the single categories from data shown in (**B**). Statistical comparisons were performed via one-

way parametric ANOVA (with Tukey's multiple comparison's test). Normality was assessed via Kolmogorov-Smirnov test. The GALT sub-score did not pass the normality test, and statistical comparisons were performed with a nonparametric Kruskal Wallis test (Dunn's Multiple Comparisons) (** $p < 0.01$; *** $p < 0.001$).

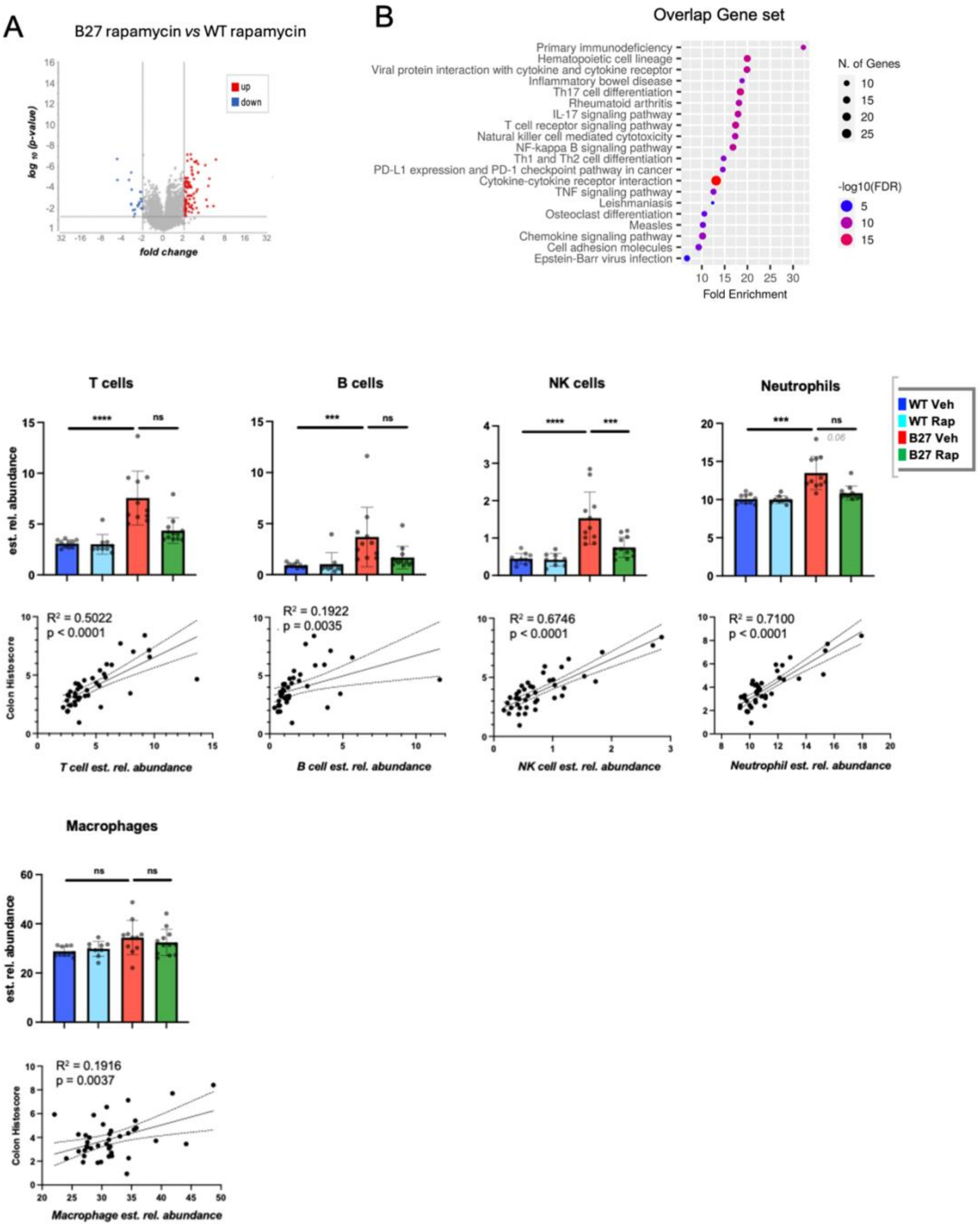

Supplemental Figure 2. Rapamycin treatment affects the transcriptome of B27-Tg rats and is associated with reduced immune cell infiltration. (**A**) Volcano plots showing differential expression of distal colon genes between rapamycin-treated B27-Tg and rapamycin-treated WT control. An ANOVA statistical test was performed on log-2 transformed RPKM values, comparing the two treatment groups. Genes that are significantly differentially expressed ($p < 0.05$ and $|FC| > 2$) between the two conditions

are indicated on each plot (underexpressed (blue), overexpressed (red). (**B**) Pathway analysis of genes that are downregulated upon rapamycin treatment in HLA-B27-Tg colon tissue compared to B27 vehicle samples using Metascape. (**C**) Immune cell abundance was estimated based on transcript expression of select genes using ImSig. Correlation plots were performed between colon histological score and estimated immune cell type abundance. R^2 and p values are as denoted on graph. Significance was determined via unpaired t-test. (* $p < 0.05$).

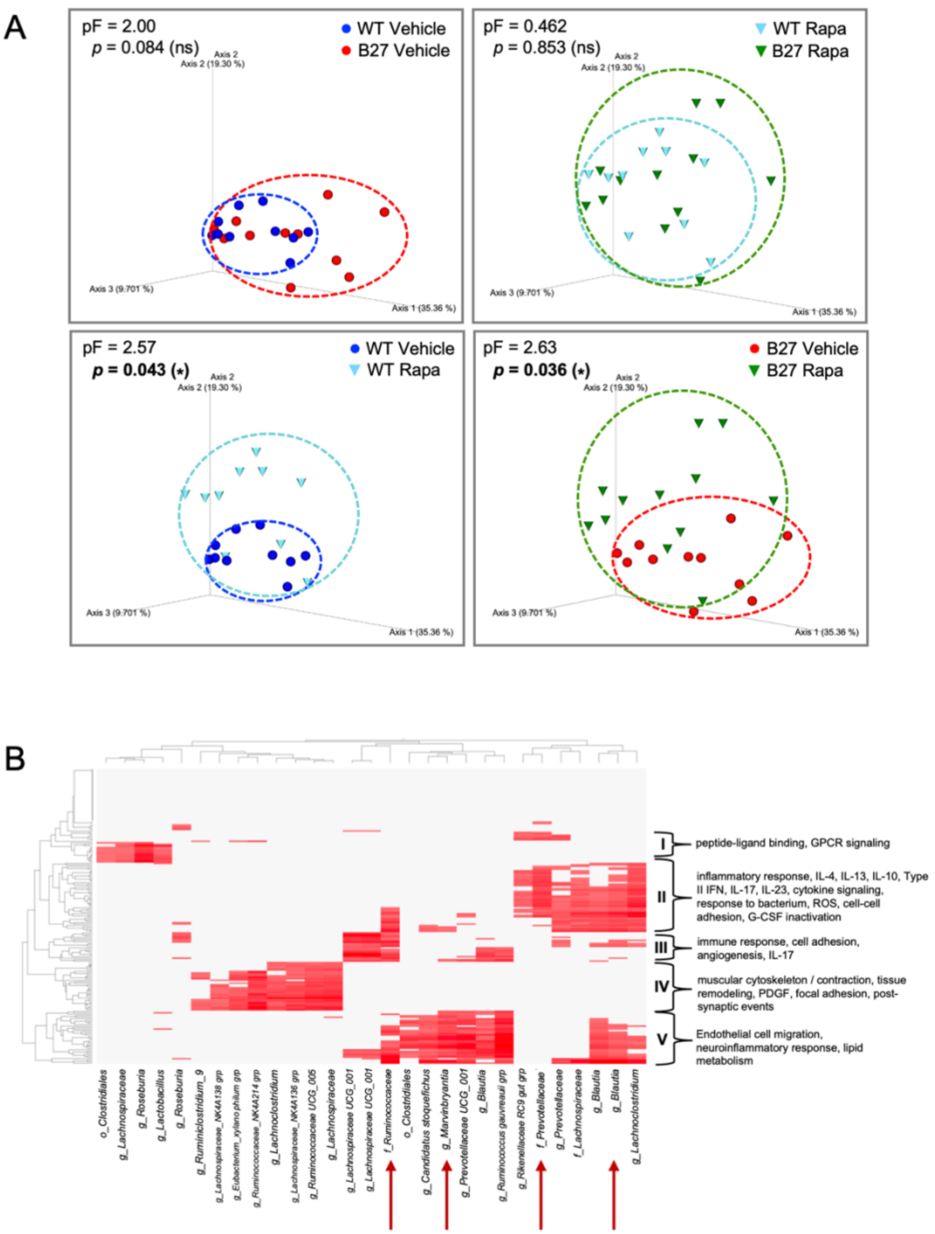

Supplemental Figure 3. Effects of treatment on gut microbial composition. (**A**) Microbial abundance in the cecal lumen was assessed for via 16S rRNA sequencing. Principle Coordinate Analysis (PCoA) plots of microbial composition (beta diversity) are shown for several comparisons (all four groups are shown together in Fig. 4C). Each data point represents a cecum lumen sample from a single animal. The data distribution of each treatment group is shown by ellipsoids. Statistical comparisons were performed via weighted UniFrac Permanova. Test statistics including pseudo-F score (pF) and p value

are as indicated in the figures. A greater pseudo-F value corresponds to a greater degree of cluster separation. (**B**) Heat map of correlations (r) between the relative frequency of cecum lumen microbial OTUs and host cecum transcript expression. Cecum lumen collected from animals at experimental endpoint was assessed for microbial abundance via 16S rRNA sequencing. A Pearson correlation was performed between sample microbial relative frequency and transcript gene expression. Microbe-transcript pairs with significant correlations ($p < 0.001$), microbes correlating with at least 5% of total transcripts, and gene transcripts correlating with at least 2.5% of microbes are shown. The resultant correlated pairs were then subjected to hierarchical clustering. Microbial OTUs (columns) and host cecal gene transcripts (rows) are as annotated on the heat map. Microbe-transcript pairs with a positive correlation are shown in red on the heat map, with a stronger correlation coefficient (r) value corresponding to a deeper red hue. Microbe-transcript pairs that were negatively correlated are shown in blue; however, no negatively correlated pairs were identified. Metascape and Toppgene pathway analysis was performed on gene transcripts within indicated clusters, with pertinent results annotated on the righthand corner of the heatmap. Metascape results are shown in Supp. Fig 2B.

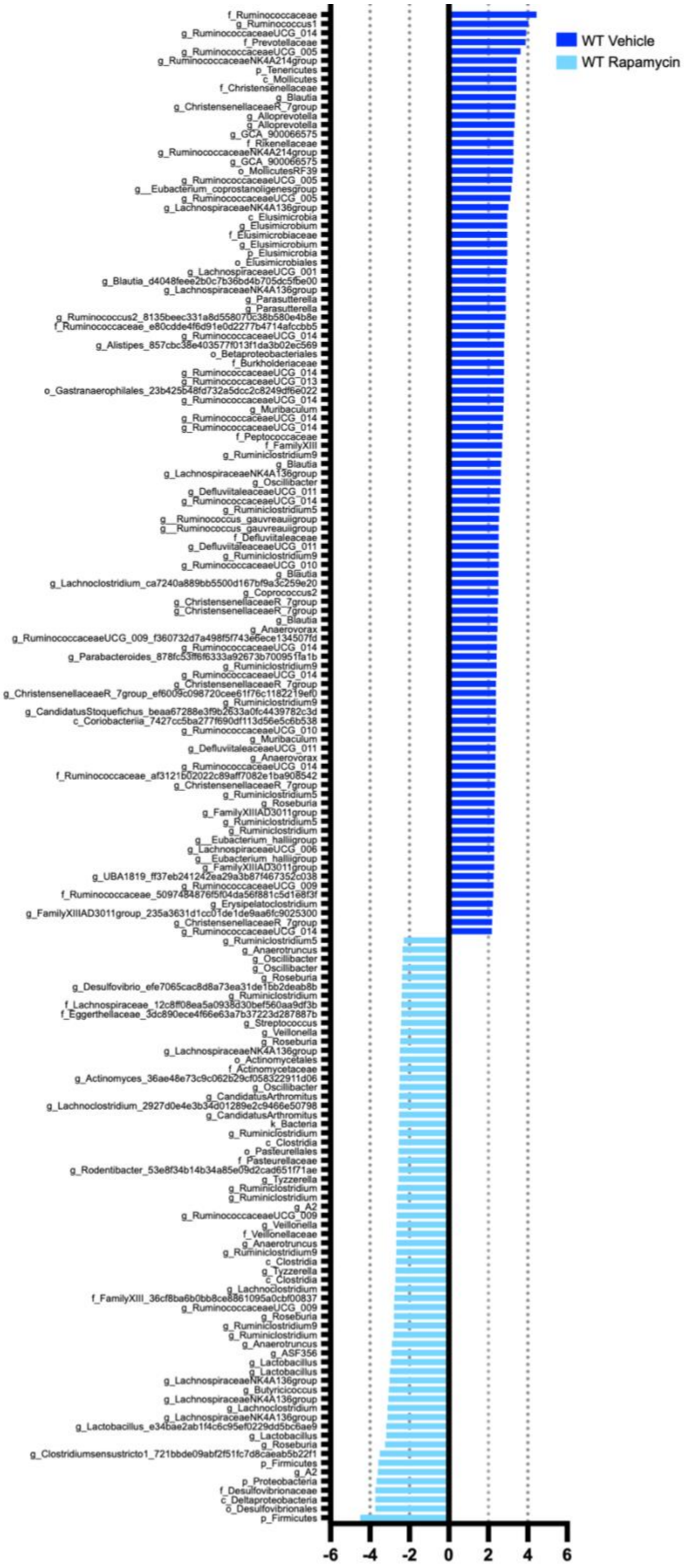

Supplemental Figure 4. Rapamycin treatment alters the microbiome in the absence of inflammation in WT animals. Cecum lumen samples were assessed for microbial

abundance via 16S rRNA sequencing. Differential abundance was assessed via LefSe analysis. Significantly differentially abundant (| LDA | > 2, $p < 0.05$) Operational Taxonomic Units (OTUs) are shown. Microbial OTUs that are more abundant in WT vehicle-treated samples as compared to WT rapamycin-treated samples are shown as dark blue bars. Microbial OTUs that are more abundant in WT rapamycin-treated samples compared to WT vehicle-treated as shown as light blue bars.
